# Development of a Powder Analysis Procedure Based on Imaging Techniques for Examining Aggregation and Segregation Phenomena

**DOI:** 10.3390/jimaging10030053

**Published:** 2024-02-21

**Authors:** Giuseppe Bonifazi, Paolo Barontini, Riccardo Gasbarrone, Davide Gattabria, Silvia Serranti

**Affiliations:** 1 Department of Chemical Engineering, Materials and Environment, Sapienza University of Rome, via Eudossiana 18, 00184 Rome, Italy; davide.gattabria@uniroma1.it (D.G.); silvia.serranti@uniroma1.it (S.S.); 2 Research Center for Biophotonics, Sapienza University of Rome, Corso della Repubblica 79, 04100 Latina, Italy; 3 CHEMI S.p.A., Via Vadisi 5, 03010 Patrica, Italy; p.barontini@italfarmacogroup.com; 4 Research and Service Center for Sustainable Technological Innovation (Ce.R.S.I.Te.S.), Sapienza University of Rome, 04100 Latina, Italy; riccardo.gasbarrone@uniroma1.it

**Keywords:** powder, image analysis, fractal dimension, segregation, aggregation, particles, quality control

## Abstract

In this manuscript, a method that utilizes classical image techniques to assess particle aggregation and segregation, with the primary goal of validating particle size distribution determined by conventional methods, is presented. This approach can represent a supplementary tool in quality control systems for powder production processes in industries such as manufacturing and pharmaceuticals. The methodology involves the acquisition of high-resolution images, followed by their fractal and textural analysis. Fractal analysis plays a crucial role by quantitatively measuring the complexity and self-similarity of particle structures. This approach allows for the numerical evaluation of aggregation and segregation phenomena, providing valuable insights into the underlying mechanisms at play. Textural analysis contributes to the characterization of patterns and spatial correlations observed in particle images. The examination of textural features offers an additional understanding of particle arrangement and organization. Consequently, it aids in validating the accuracy of particle size distribution measurements. To this end, by incorporating fractal and structural analysis, a methodology that enhances the reliability and accuracy of particle size distribution validation is obtained. It enables the identification of irregularities, anomalies, and subtle variations in particle arrangements that might not be detected by traditional measurement techniques alone.

## 1. Introduction

A particle size distribution (PSD), which indicates the proportion of particles at different sizes within a sample, provides detailed information about the characteristics and properties of materials [1]. Accurate analysis of PSD plays a significant role in various fields, including materials science, pharmaceuticals, mining, environmental monitoring, and manufacturing processes. By examining the PSDs, scientists and researchers can gain valuable insight into the behavior and performance of materials.

In the pharmaceutical industry, particle size analysis is crucial for ensuring the quality and efficacy of drug formulations. The PSD directly affects the therapeutic effect of the drug, as particle sizes can impact dissolution rate, bioavailability, and absorption. Moreover, particle sizes influence production processes and the quality of the final product, with uniform particles aiding in mixing, compression, and formulation [2]. Inconsistent particle sizes can lead to stability issues, difficulties in filling capsules, and incorrect dosage. Additionally, PSD affects compatibility with other excipients and formulations, as well as the mode of drug administration.

In the pharmaceutical sector, particle size evaluation is crucial for quality control purposes, ensuring consistency and compliance, and identifying manufacturing variations or issues for timely interventions [3]. Moreover, by studying the PSD of a specific bulk material and/or powder, researchers can develop optimized drug delivery systems (DDSs) and enhance the therapeutic effectiveness of medications [4].

In this context, the development and application of image analysis procedures for the analysis of PSD can help provide precious information for optimizing industrial processes (i.e., quality and performance of various substances and products).

Most of the commercially available techniques for particle size measurement are designed for the analysis of dispersed particles [5]. In this scenario, sieving, an approach that is well-suited to bulk powders, is acknowledged for its simplicity but is time-consuming when analyzing particle blends [6]. Laser diffraction, extensively applied for particle size measurement, involves dispersing powder in a fluid, allowing a laser diffraction device to effectively average various dimensions as particles flow randomly through the light beam [7,8]. Other methods, such as dynamic light scattering [9], focused-beam reflectance measurement (FBRM) [10], acoustic spectroscopy [11,12], and near-infrared spectroscopy (NIRS) [13], have also proven successful in particle size measurement. However, many of these techniques are not designed for the rapid and noninvasive analysis of bulk powders, requiring individual particle dispersion (i.e., in a liquid or gas) before analysis. In this context, computer-assisted microscopy or traditional microscopy is frequently regarded as a standard method in particle size analysis [14]. Visual and microscopic inspection plays a crucial role in understanding materials. Although microscopy and image analysis (IA) offers fast and versatile approaches, sample preparation and powder sample dispersion remain challenges in routine IA. Consequently, exploring novel approaches that leverage image information for particle size and shape analysis is a subject still worth studying and developing.

In this article, the proposed methods, which focus on utilizing advanced image analysis techniques, both fractal and texture-based, aim to investigate a dedicated fast and noninvasive quality control approach to bulk powder. This qualitative approach is designed to provide useful information about the PSD of bulk powders and determine particle aggregation and/or segregation phenomena. In this context, fractal analysis allows for the quantification of irregularities and self-similar patterns within particle distributions, thus enabling a more precise and detailed understanding of particle size variations and spatial arrangement [15]. The fractal analysis also allows for the identification of fractal dimensions and spatial correlations within particle distributions, aiding in the differentiation between aggregation and segregation behaviors. Textural analysis contributes to characterizing patterns and spatial correlations, as detectable in particle images [16]. In more detail, the approach presented in this paper was designed to investigate a series of Out-of-Trend (OoT)/Out-of-Specification (OoS) instances regarding the specified PSD of the final API (Active Pharmaceutical Ingredient) product, which were encountered throughout the production of 2021 in a manufacturing site of an Italian pharmaceutical chemical company.

Initial quality control (QC) internal investigation did not conclusively identify the primary cause behind these quality deviations. The company’s QC team suspected the presence of at least one systemic phenomenon that could potentially impact either the analytical results (prompting an investigation into their accuracy by testing products using an alternative technique to light scattering) or the actual product quality itself. In the case of a definitive resolution regarding the analytical results, the focus would have shifted toward identifying more effective routes to guide the internal investigation.

**Figure 1:**
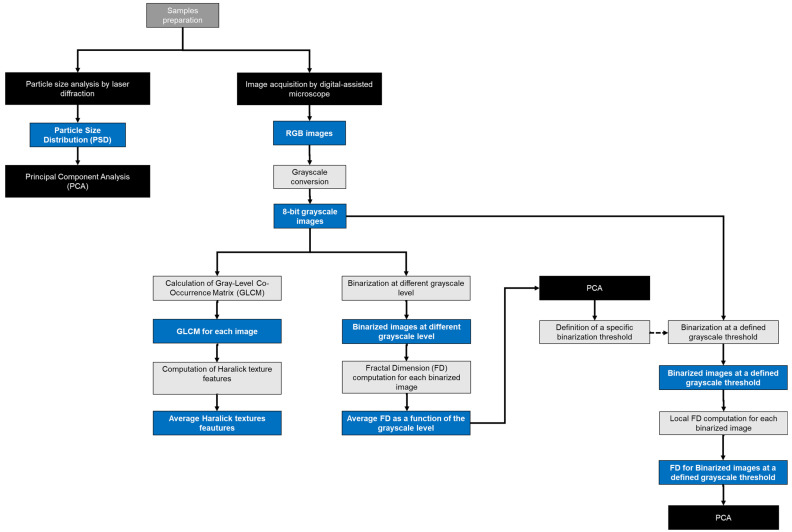
Schematic diagram of the methodological approach.

The methodology presented in this paper involves several steps, as shown in Figure 1. First, the sample is prepared by dispersing the powder on a suitable sample holder. The dispersed sample is then placed on a microscope slide. Next, a microscope equipped with a high-resolution camera captures images of the sample, ensuring that a representative portion of the sample is analyzed. Once the images are obtained, image processing algorithms are applied to perform fractal analysis and textural analysis.

Following this approach, researchers and manufacturers can gain deeper insights into the characteristics of their samples (i.e., bulk pharmaceutical solids). The method enables the identification of samples with a broad particle size distribution, indicating the presence of particles spanning a wide range of sizes. It also helps to detect samples with two distinct particle size distributions, which may indicate specific phenomena such as particle aggregation or segregation.

## 2. Materials and Methods

### 2.1. Sample Preparation and Digital Images Acquisition

**Figure 2:**
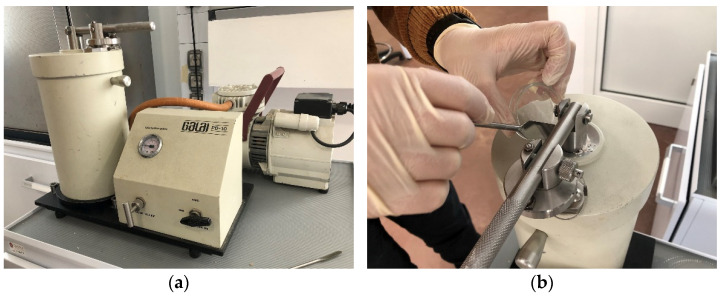
GALAI PD-10 vacuum gauge (**a**) and sample dispersion on the device’s chamber (**b**).

The method presented in this paper is based on an accurate sample preparation procedure using the PD-10 powder dispersing system (Galai Production Ltd., Midgal Haemek, Israel). This system ensures uniform dispersion of the powder on a sample holder by activating a lever after creating a vacuum within the chamber. First, the powder is placed on top of the chamber. The device is then activated to ensure a vacuum in the chamber. By activating the lever, the powder is finely dispersed onto a glass slide, which will later allow for microscopic observation. This preliminary step of sample preparation is shown in Figure 2.

**Figure 3:**
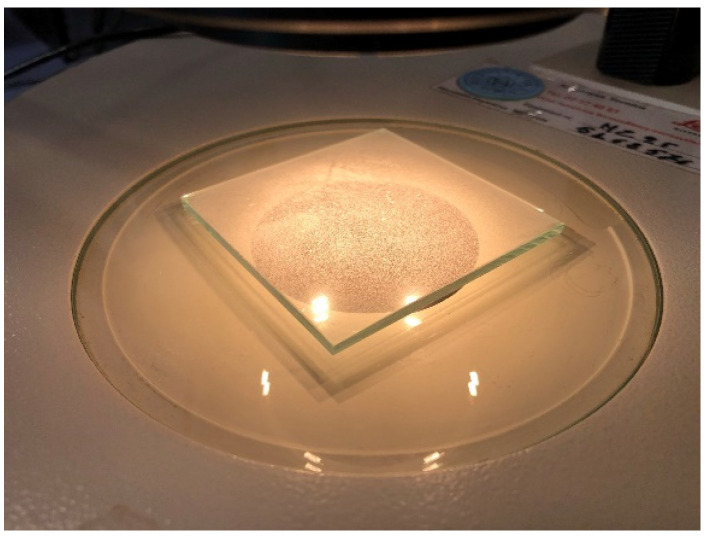
Close-up of the glass slide, as resulting after powder dispersion, in order to be analyzed under transmitted light by the Leica Wild MZ95™ microscope.

The glass slide (Figure 3) can be then analyzed by a computer-assisted microscope in transmitted light. Image acquisition was performed by a Wild MZ95™ microscope (Leica AG, Wetzlar, Germany) equipped with the TOUPCAM™ U3CMOS Series (ToupTek Photonics, Hangzhou, China) digital camera (complementary metal–oxide–semiconductor, CMOS, camera), directly interfaced with a Personal Computer (Figure 3).

### 2.2. Implementation of MATLAB Algorithms for Image and Extracted Data Analysis

Images and data have been processed using MATLAB (Ver 9.12., MathWorks, Natick, MA, USA). The processing involves several procedures, including filter application, color balancing, binarization, and segmentation.

In detail, MATLAB routines were developed to process both the acquired digital microscope images in batches and the data resulting from their analyses. The operative steps of these routines are reported in the following.

*Routine 1—Fractal dimension as a function of the grayscale level used as the binarization threshold.*

Operative steps:

- Conversion of the original image to grayscale.
- Binarization of the grayscale image at different thresholds for the grayscale levels, ranging from 0 to 255 with a step of 5.
- Calculation of the fractal dimension using the box-counting method for each binarized image.
- Obtaining the curve of the fractal dimension as a function of the binarization threshold (grayscale level) of the image.

The MATLAB scripts and functions utilized for fractal dimension, as a function of the grayscale level used as the binarization threshold adopted on multiple images, are available at GitHub: https://github.com/Gasby90/FD_multiple_images_grayscale_level (accessed on 20 November 2023). 

*Routine 2—Calculation of Haralick texture features:*

Operative steps:

- Conversion of the original image to 8-bit grayscale.
- Calculation of the co-occurrence matrix in 4 directions (0°, 45°, 90°, 135°).
- Calculation of Haralick parameters for the 4 directions.
- Calculation of the averaged Haralick parameters for the 4 directions.

The MATLAB script used to perform textural analysis on multiple images is available at GitHub: https://github.com/Gasby90/textural_multiple_images (accessed on 20 November 2023). 

**Fractal dimension (box-counting method) calculation.** Fractal dimension (FD) is a statistical measure indicating how completely a fractal fills space [17,18]. A fractal is a geometric object with internal self-similarity: it repeats its shape in the same way at different scales so that enlarging any part of it produces a figure similar to the original [19].

In fractal geometry, the Minkowski-Bouligand dimension, also known as the Minkowski dimension or box-counting dimension, is a way to determine the fractal dimension of a set S in Euclidean space $R^{n}$, or more generally, in a metric space (X, d) [20,21,22]. To calculate this dimension for a fractal set *S*, it is assumed that this fractal lies on a uniformly spaced grid. The measure of cell size is determined by observing how this number changes as the grid becomes finer. Let The box-counting dimension is defined by:

(1) $${dim}_{box}\left(S\right)≔\underset{r\to 0}{\lim}\frac{log(n\left(r\right))}{log(1/r)}$$

where n(r) is the number of boxes of side length ε needed to cover the set.

If the limit does not exist, it is still possible to compute the upper and lower limits to determine the upper and lower box dimensions, respectively. The upper box dimension is sometimes referred to as the Kolmogorov or entropy dimension, while the lower box dimension is also known as the lower Minkowski dimension.

Box counting is a method that is preferred due to its simplicity and robustness, making it easily applicable to a wide range of shapes [23].

In the described methodology, we adopted the boxcount.m [24] MATLAB function (Appendix B), which is implemented to determine the fractal properties of the two-dimensional binarized images.

The utilized algorithm operates as follows:

1. The binarized image is divided into square boxes of size *r* × *r*. The number of boxes (n(r)) containing a portion of the shape is counted;
2. The process starts with the smallest box that encompasses the entire binarized shape and has a side length of 2 times the pixel size. This ensures that the entire shape is covered by the initial box;
3. The process is then repeated, with the box size (*r*) being divided by 2 at each iteration. This is continued until the box size reaches the pixel size of the image. Each iteration counts the number of boxes that contain a portion of the shape.

The fractal dimension is finally determined by calculating the slope of the line obtained from the linear regression of the log(n(r)) values against log(1/r). The absolute value of the slope represents the fractal dimension of the shape in the 2D image. The slope indicates how the number of boxes changes with respect to the box size, providing insights into the complexity and self-similarity of the shape.

**Gray-Level Co-occurrence Matrix and Haralick texture features calculation.** The Gray-Level Co-occurrence Matrix (GLCM) is a matrix defined on an image that captures the distribution of co-occurring pixel values (grayscale or color values) at a given offset [25].

The GLCM is defined for an image I of size n×m, parameterized by an offset (Δx, Δy), by the equation:

(2) $$C_{\Delta x,\Delta y}(i,j)=\sum_{p=1}^{n}\sum_{q=1}^{m}\left\{\begin{array}{c} 1,\;if\;I(p,q)=i\;and\;I(P+\Delta x,\;q+\Delta y)=j\\ 0,\;otherwise \end{array}\right.$$

A GLCM evaluates the texture characteristics occurring in an image. Texture encompasses the geometric patterns and/or repetitive arrangements of gray levels found within an image. When a region within an image exhibits a consistent texture, it implies that certain local statistical measures or other properties remain steady, change gradually over time, or display an approximate periodicity. Due to the typically large and sparse nature of co-occurrence matrices, it is common to employ various metrics from the matrix to derive a more meaningful set of features, commonly known as the Haralick texture features [26].

The HaralickTextureFeatures.m [27] MATLAB function (Appendix B) was adopted to calculate GLCM for each image and extract specific Haralick texture features. The GLCM is calculated on the basis of the frequency with which a pixel with intensity (gray level) i is found in a specific spatial relationship with a pixel with intensity j. By default, the spatial relationship is defined as the pixel of interest and the pixel immediately to its right (horizontally adjacent). Each element (i,j) in the resulting GLMC is simply the sum of the number of times the pixel with value i has been observed in the specified spatial relationship with a pixel with value j in the input image. The number of gray levels in the image determines the size of the GLCM matrix. GLCMs are calculated for the four directions: 0° (offset: [0 D]), 45° (offset: [−D D]), 90° (offset: [−D 0]), and 135° (offset: [−D D]).

Finally, the texture features are calculated from the resulting GLMC according to Haralick’s work [26,28].

The Haralick texture features extracted from GLCM are Angular Second Moment (Energy), Contrast, Correlation, and Entropy. These features are briefly described below.

*Angular Second Moment (Energy)*

The angular second moment (ASM), also referred to as energy, serves as an indicator of local intensity variation or contrast within an image. It can be mathematically represented by the equation:

(3) $$ASM=\sum_{i}\sum_{j}{\left\{p(i,j)\right\}}^{2}$$

where p(i,j) represents the (*i, j*)-th element in a grayscale spatial dependence matrix that has been normalized (=P(i,j)/R).

The ASM provides insights into the overall regularity of an image, taking into account the differences between adjacent pixels. In an organized image, where value pairs occur more frequently, each value p(i, j) from the co-occurrence matrix is assigned a weight. This weight increases with higher levels of order and decreases with increased disorder within the image.

*Contrast.*

The feature Contrast (CON) quantifies the difference between the gray levels of a pixel and its surrounding region and is defined by the equation:

(4) $$CON=\sum_{n=0}^{N_{g-1}}n^{2}\left\{{\sum_{i=1}^{N_{g}}\sum_{J=1}^{N_{g}}p(i,j)}_{\left|i-j\right|=n}\right\}$$

where $N_{g}$ denotes the number of distinct gray levels present in the image.

An absence of any contrast (*CON* = 0) occurs when the values of i and j are the same, indicating that the pixels being compared are entirely like their surroundings, then the weight $n^{2}$ in the CON function is n=|i−j|=0. In the case where i and j differ by 1, a small contrast exists, and the weight $n^{2}$ is assigned a value of 1. As the difference between  i and j increases to 2, the contrast intensifies, and the corresponding weight grows to $n^{2}$ = 4. The weight $n^{2}$ exhibits an exponential increase with the widening discrepancy in gray levels between the pixel values i and j.

*Correlation.*

The feature Correlation (COR) captures the linear relationship between gray levels of neighboring pixels and is defined by the following equation:

(5) $$COR=\frac{\sum_{i}\sum_{j}\left(ij\right)p\left(i,j\right)-\mu_{x}\mu_{y}}{\sigma_{x}\sigma_{y}}$$

where $\mu_{x}$, $\mu_{y}$ and $\sigma_{x}$, $\sigma_{y}$ are the mean and standard deviation of $p_{x}$ and $p_{y}$, respectively. $p_{x}(i)$ represents the *i*-th element in the marginal probability matrix, obtained by summing the rows of $p\left(i,j\right)$, given by $\sum_{j=1}^{N_{g}}P(i,j)$ and $p_{y}\left(j\right)$ signifies the sum of probabilities for all *i*-th elements in column *j*, computed as $\sum_{i=1}^{N_{g}}P(i,j)$.

A high correlation between two pixels implies a strong predictability of their association, as expressed through a linear regression equation. Generally, pixels exhibit a greater tendency to be correlated with nearby pixels compared to those located further away. This property can be leveraged to estimate the size of an object within the analyzed image. In the implemented code, a condition is implemented to check the values of $\sigma_{x}$ and $\sigma_{y}$: if either of them equals zero, the correlation is set to 1.

*Entropy.*

In this context, Entropy (ENT) represents the opposite of energy and, therefore, measures the level of disorder in the image.

(6) $$ENT=-\sum_{i=1}^{N_{g}}\sum_{j=1}^{N_{g}}p(i,j)\cdot log\;p(i,j)$$

Each value p(i, j) within the co-occurrence matrix always falls within the range of 0 and 1, as the matrix is represented in the form of probabilities. The logarithm of this quantity will consistently yield a result of either 0 or a negative value. When the p(i, j) value decreases (indicating a lower likelihood of that specific pixel combination occurring), the absolute value of log p(i, j) increases, subsequently raising the entropy value. The negative sign preceding the function ensures that each term becomes positive.

**Exploratory analysis by Principal Component Analysis.** In the field of multivariate statistics, Principal Component Analysis (PCA) is a technique used for data simplification and analysis [29,30]. In fact, PCA is typically the first step when dealing with complex multivariate data. PCA is a powerful tool for exploratory data analysis, allowing the evaluation of an entire data matrix to describe the structure of samples and variables involved, as well as to assess potential correlations between samples and analyzed variables.

In the case study, PCA was adopted as an exploratory analysis technique for:

- PSDs of the analyzed samples.
- Fractal dimension curves as a function of the grayscale level are used as the binarization threshold for the samples.
- Fractal dimension curves are a function of the counting box size with a defined threshold for the samples.

PCA was performed in MATLAB environment using the PLS_toolbox (Ver. 9.0; Eigenvector Research Inc., Manson, DC, USA).

The data were pre-processed using the Autoscale algorithm before performing PCA. Autoscale is a commonly used pre-processing method that involves centering each column (variable) by subtracting its mean and dividing it by the standard deviation of that column.

### 2.3. Method Testing and Validation

The samples used in this study were provided by CHEMI S.p.A. (Frosinone, Italy). 

The analyzed samples were coded according to their product requirement compliance (i.e., PSD specifications) from the same batch clustered into two categories, which are ‘In Specification’ (IS) (in case of complete overlapping with the product specifications) and ‘Out of Specification’ (OOS) (in case of small or great deviation). 

The IS samples are “aligned” with the particle size distribution specifications and meet the requirements. On the other hand, the OOS samples show a PSD shift toward higher values. Simultaneously, these samples exhibit the presence of a portion of material characterized by a “correct” particle size distribution and another portion characterized by a particle size distribution representing larger particles (i.e., bimodal distribution). The presence of these samples can result in greater heterogeneity within the batch and, in particularly severe cases, lead to a tendency for segregation between the two components.

The IS samples are labeled as A0802, A0804 and A0801. The Identifications (IDs) of OOS samples are A0065, A0418 and A0053.

**Figure 4:**
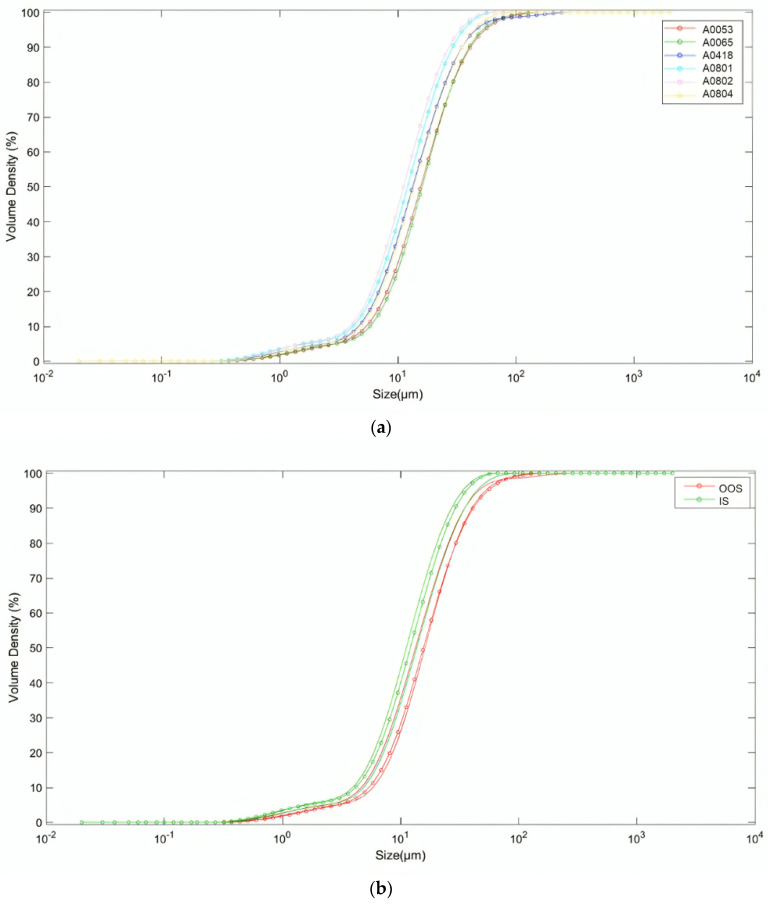
Particle size distribution of the samples labeled according to sample ID (**a**) and product requirement compliance (i.e., ‘Out of Specification’, OOS, or ‘In Specification’, IS, categories) (**b**). The samples labeled A0065, A0418, and A0053 correspond to the OOS category, while A0801, A082, and A0804 correspond to the IS category. Particle size distributions (PSDs) were obtained by laser diffraction. At the same particle size, the IS samples generally exhibit a lower volume density compared to the OOS samples. PSD curves of the OOS samples are slightly shifted to the right (or skewed) compared to the PSD curves of the IS samples.

This heterogeneity in particle size distribution is clearly visible in the curves obtained from a laser diffraction particle size analyzer (Malvern Instruments Ltd., Malvern, Worcestershire, UK), as shown in Figure 4, with particle sizes ranging from 0.32 µm to 285.73 µm (100% passing). The percentiles of the PSDs obtained by the laser diffraction method are reported in Appendix A. The laser diffraction method is based on the scattering of laser light by particles [31]. This methodology relies on two optical models: the Fraunhofer diffraction model and the Mie theory. The Fraunhofer model assumes a parallel laser beam and a distant detector relative to the particle size, providing an approximation of particle size distribution. Conversely, the Mie theory, rooted in Maxwell’s equations, offers a more precise solution by considering electromagnetic wave propagation and other phenomena beyond diffraction. This theory requires knowledge of the material’s refractive index and provides an exact solution for light scattering from homogeneous spheres. Both models output volume-based PSD, which characterizes PSD in terms of volume rather than mass.

The analyzed product, in its hydrochloride form, precipitates from a solution by adding NaOH, which, depending on the rate of addition, results in obtaining particles of the correct size. If the NaOH is added within a certain time frame (about 45–60 min), a PSD curve that meets the compliance criteria is achieved. However, if the NaOH is added after 45–60 min, the particles tend to aggregate, posing the risk of deviating the product from specifications, even after routine milling with a Fitzpatrick mill. In this case, the average PSD of the samples is different due to increased heterogeneity and segregation of particles. There are also other factors that have a significant impact on the PSD, i.e., the use of a rotating paddle dryer and the portion of the product that is subjected to mechanical action. Depending on the yield, moisture content, loading methods, etc., the distribution of the wet product inside the dryer does not always occur uniformly, nor in a way that ensures the paddles act on the entirety of the product. The portion of the product that does not undergo the action of the paddles will have a coarser PSD compared to the portion that does experience the full effect. This leads to two problems: (i) the “average” PSD is shifted towards higher values and, in extreme cases, may exceed the specification (which requires the entirety of the sample to be below 100 µm), and (ii) the presence of a fraction with the correct PSD alongside a coarser portion, causing overall batch non-uniformity. In severe instances, this discrepancy might prompt the segregation of the two components, accurately identified by the internal quality system, therefore preventing the release of affected batches.

Utilizing fractal dimension and textural features provides a comprehensive insight into the PSD of the powder product. These advanced imaging techniques enable a more nuanced understanding of their spatial arrangement and irregularities, which may not be fully captured by the laser diffraction technique that assumes that the samples are formed by perfect spherical particles. By examining textural characteristics and fractal dimensions at different grayscale levels, valuable insights into particle aggregation and segregation phenomena can be gained despite operating within a two-dimensional space.

### 2.4. Sample Preparation and Image Acquisition

In the initial tuning phase of the acquisition conditions, numerous tests were carried out to choose the appropriate weight to be placed on the microscope slide. Once the appropriate weight of the product was determined for subsequent microscope analysis, each sample was weighed and dispersed onto the slide using the powder dispersing system. The dry powder samples were dispersed within the vacuum dispersion chamber of the Galai PD-10 at a pressure of −85 kPa (equivalent to −0.85 bar as indicated on the pressure gauge). This negative pressure facilitates the intake of the powder and its dispersion on the glass slide. After preparing the slide for analysis, digital images were acquired using the microscope. During this phase, two sets of images were acquired with the Wild MZ95™ microscope, which are:

- seven images at three different magnifications (×1, ×3.2, ×5) for two samples (one OOS and one IS, the N series samples as reported in Table 1) and
- ten images for each sample at a magnification of ×1 (Table 2).

The weight range for the analysis tests at different magnifications (×1, ×3.2, and ×5) on samples A0801 and A0065 (N Series) is 0.06–0.08 g. In more detail, the N series comprises a subgroup consisting of two samples, subsampled from A0065 (OOS) and A0801 (IS): N_A0065 and N_A0801. Meanwhile, the weight range used for the microscope analysis at magnification ×1 is 0.14–0.17 g. The weight of each sample dispersed on the slide and analyzed is reported in Table 1 and Table 2.

## 3. Result and Discussion

### 3.1. Principal Component Analysis Applied to Particle Size Distributions

**Figure 5:**
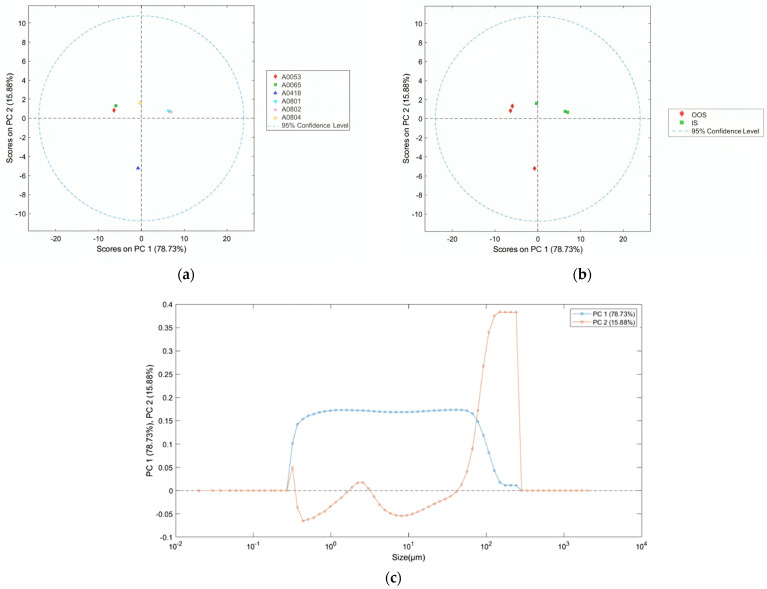
PCA scores plot (PC1 vs. PC2) of the particle size distribution for samples (**a**) and conditions (**b**) and PCA loadings plot (**c**). The 95% confidence level reported in the PCA scores plot represents the statistical certainty regarding the dispersion of data points around their respective centroids or means.

The results of the PCA applied to the particle size distribution curves of the analyzed samples are shown in Figure 5. As previously defined, the particle sizes of the samples range from 0.32 µm (non-zero pass) to 285.73 µm (100% pass). As can be observed from the scores plot of the first two principal components (Figure 5a,b), the IS samples are primarily located in the positive space of PC1 and PC2 (due to the ranges 0.26–0.34 µm, 1.7–3.0 µm, 42–290 µm). 

However, sample A0804 (IS) is situated in the negative space of PC1 and the positive space of PC2. On the other hand, the OOS samples are primarily located in the negative space of PC1 and the positive space of PC2. Sample A0418 (OOS) is situated in the negative space of both PC1 and PC2. As can be observed in the PCA loadings plot (Figure 5c), PC1 is positive within the range of 0.27–300 µm. PC2 is positive within the ranges of 0.26–0.34 µm, 1.7–3.0 µm, and 42–290 µm. Finally, PC2 is negative within the ranges of 0.34–1.7 µm and 3.1–42 µm.

### 3.2. Binarization at Different Grayscale Levels

**Figure 6:**
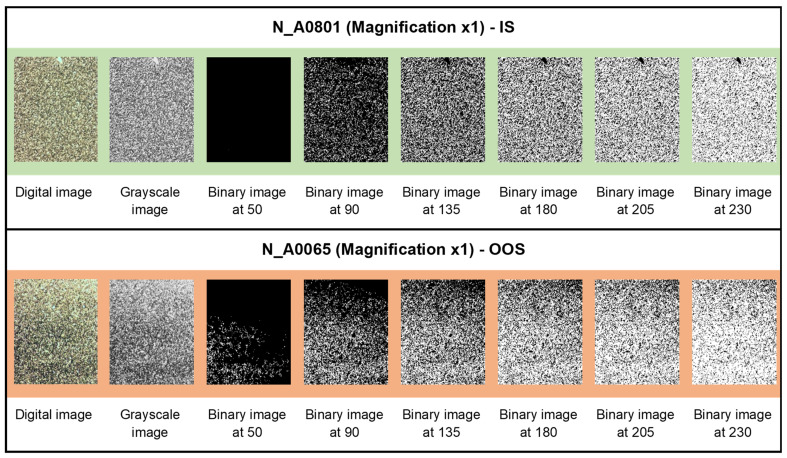
Binarization process at different grayscale levels for samples from the N series acquired at a magnification of ×1.

**Figure 7:**
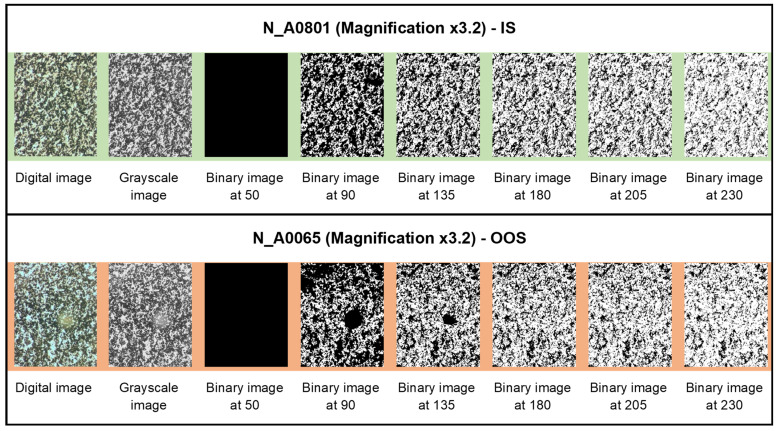
Binarization process at different grayscale levels for samples from the N series acquired at a magnification of ×3.2.

**Figure 8:**
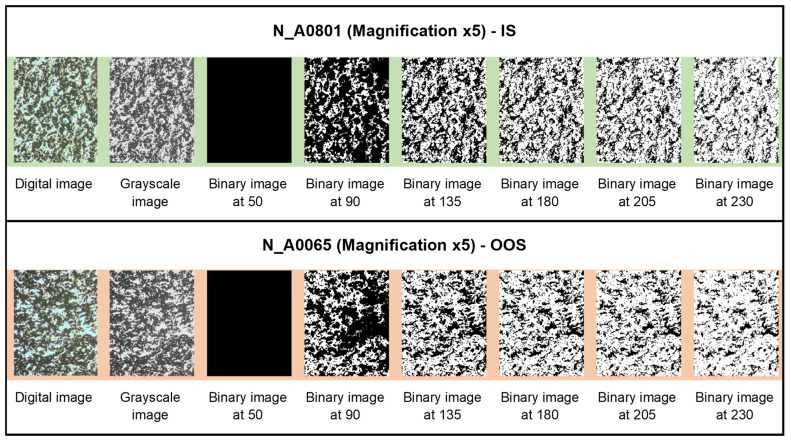
Binarization process at different grayscale levels for samples from the N series acquired at a magnification of ×5.

In Figure 6, Figure 7 and Figure 8, examples of the binarization process at different grayscale levels are shown for the samples from the N series (N_A0801—IS, and N_A0065—OOS), respectively, acquired at magnifications of ×1, ×3.2, and ×5.

### 3.3. Average Fractal Dimension as a Function of the Grayscale Level Adopted as Binarization Threshold

**Figure 9:**
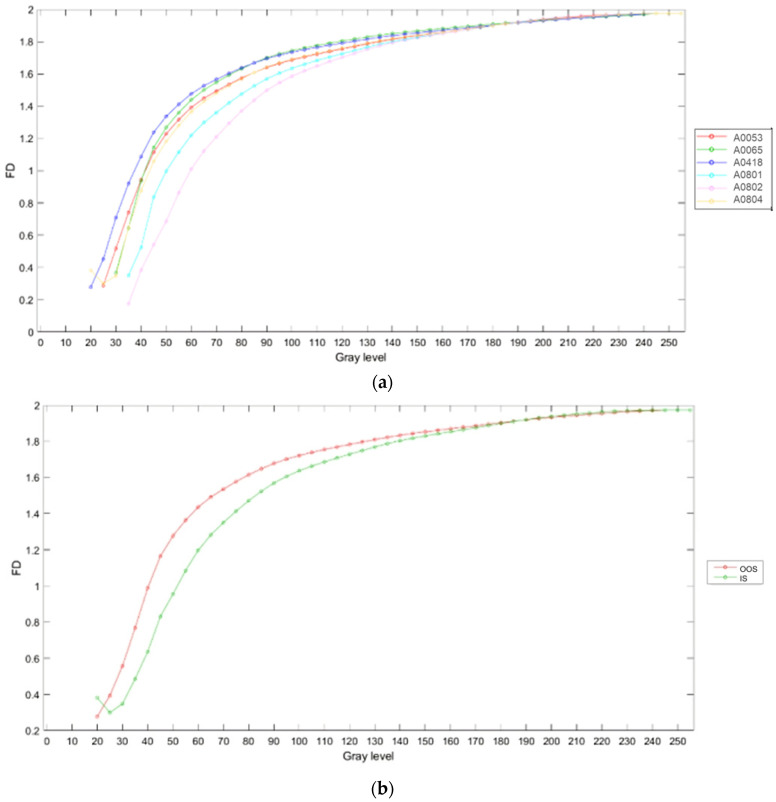
Mean fractal dimension (FD) of analyzed samples as a function of the adopted grayscale level used as binarization threshold labeled according to sample ID (**a**) and averaged according to product requirement compliance (**b**), i.e., Out of Specification (OOS) or In Specification (IS) categories. The samples designated as A0065, A0418, and A0053 fall within the OOS category, whereas A0801, A082, and A0804 are categorized as IS. At the same grayscale level, the OOS samples exhibit a higher average fractal dimension compared to the IS samples within the grayscale range of 20 to 170.

The average fractal dimension (calculated using the box-counting method, averaged over the size of the calculation boxes) and its standard deviation for samples from the N series (N_A0801—IS and N_A0065—OOS; samples from Table 1) at different magnifications (×5, ×3.2, and ×1) for six binarization thresholds (gray level: <50, <90, <135, <180, <205, and <230, as shown in Figure 6, Figure 7 and Figure 8) are presented in Table 3. As observed from Table 3, at the binarization thresholds of 50 and 90, the average fractal dimension of the IS sample is consistently lower than that of the OOS sample. For higher binarization thresholds, the fractal dimensions of the IS and OOS samples are nearly equivalent. Evaluating each sample at a magnification of ×1 (samples from Table 2), this trend becomes more evident when considering the entire range of binarization from 0 to 255 with a step of 5. The fractal dimensions (calculated using the box-counting method) as a function of the grayscale level adopted to perform the binarization for each analyzed sample at a magnification of ×1 (samples from Table 2) are shown in Figure 9. As highlighted in Figure 9b the average fractal dimension of the OOS samples is consistently higher than the fractal dimension calculated for the IS samples in the threshold intervals of <20 and <170 grayscale levels.

### 3.4. Co-Occurance Matrices and Haralick Descriptors

The Haralick descriptors (ASM, CON, COR, and ENT) obtained for the 4 directions (0°, 45°, 90°, and 135°) from the co-occurrence matrices of individual digital images at a magnification of ×1 (10 images per sample) were averaged per sample. Subsequently, the values obtained for the 4 directions were averaged (µ) for each sample. The results obtained are reported in Table 4.

The parameters obtained in Table 4 were then averaged according to their conditions: OOS and IS samples. The results obtained from the calculation of the averages are reported in Table 5. Even in this case, the values obtained for each direction were averaged together. As can be seen from Table 5, the ASM of the OOS samples is generally smaller than that of the IS samples, indicating that the images of the OOS samples are more disordered compared to the IS samples. The CON parameter of the OOS samples is, on average, larger than that of the IS samples. This suggests that there is a greater difference in grayscale tones between pixels in the images of the OOS samples compared to the IS samples. The COR parameter is generally lower for the IS samples compared to the OOS samples. This means that in the case of the OOS samples, there is a stronger linear dependence of grayscale levels between neighboring pixels in the images compared to the IS samples. The COR parameter could, therefore, serve as an indicator of particle aggregation in the OOS samples. Lastly, the ENT parameter is generally higher for the OOS samples compared to the IS samples, indicating that the images of the OOS samples exhibit greater disorder compared to the IS samples.

### 3.5. Principal Component Analysis of the Average Fractal Dimension as a Function of the Grayscale Level Adopted as the Binarization Threshold

**Figure 10:**
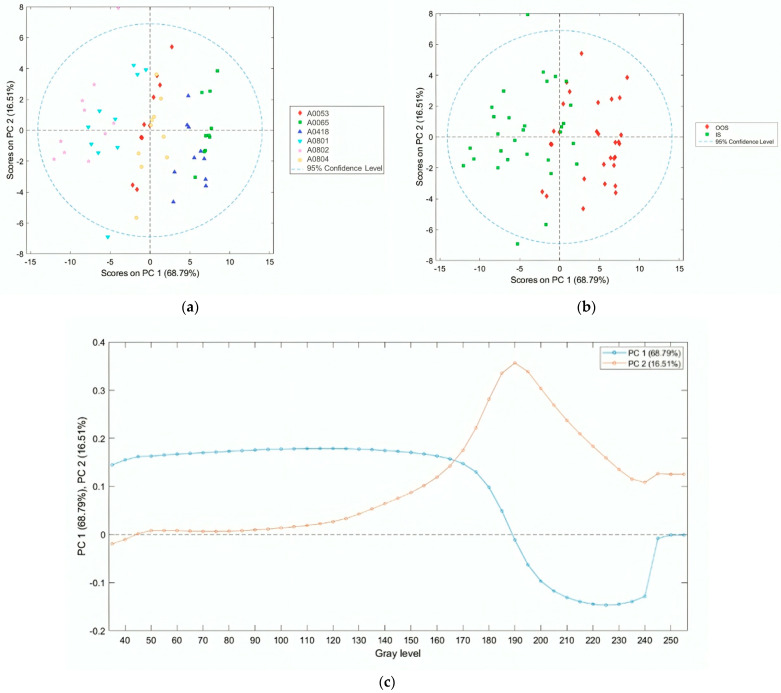
PCA scores plot (PC1 vs. PC2) of the arrays of fractal dimension (calculated using the box-counting method) as a function of the grayscale level adopted as the binarization threshold for samples (**a**) and conditions (**b**); PCA loadings plot (**c**) of the first two principal components. The 95% confidence level reported in the PCA scores plot represents the statistical certainty regarding the dispersion of data points around their respective centroids or means.

The arrays of the average fractal dimension (calculated using the box-counting method, averaged over the box size) as a function of the grayscale level adopted as the binarization threshold for each sample analyzed at a magnification of ×1 (samples in Table 2), shown in Figure 9, were modeled using PCA. The result of the PCA is summarized in Figure 10. As highlighted by the scores plot of the first two principal components, the first principal component explains approximately 70% of the variance and can discriminate between the OOS and IS groups. Specifically, the scores of the fractal dimension arrays for the OOS samples are in the positive PC1 space (from grayscale threshold 0 to 185), while those of the IS samples are in the negative space (from grayscale threshold 190 to 255). From the above, it can be concluded that for the identification of OOS samples, it is necessary to adopt a grayscale threshold <185.

### 3.6. Local Fractal Dimension with a Defined Binarization Threshold (<90 Grayscale Level) for Two Samples

**Figure 11:**
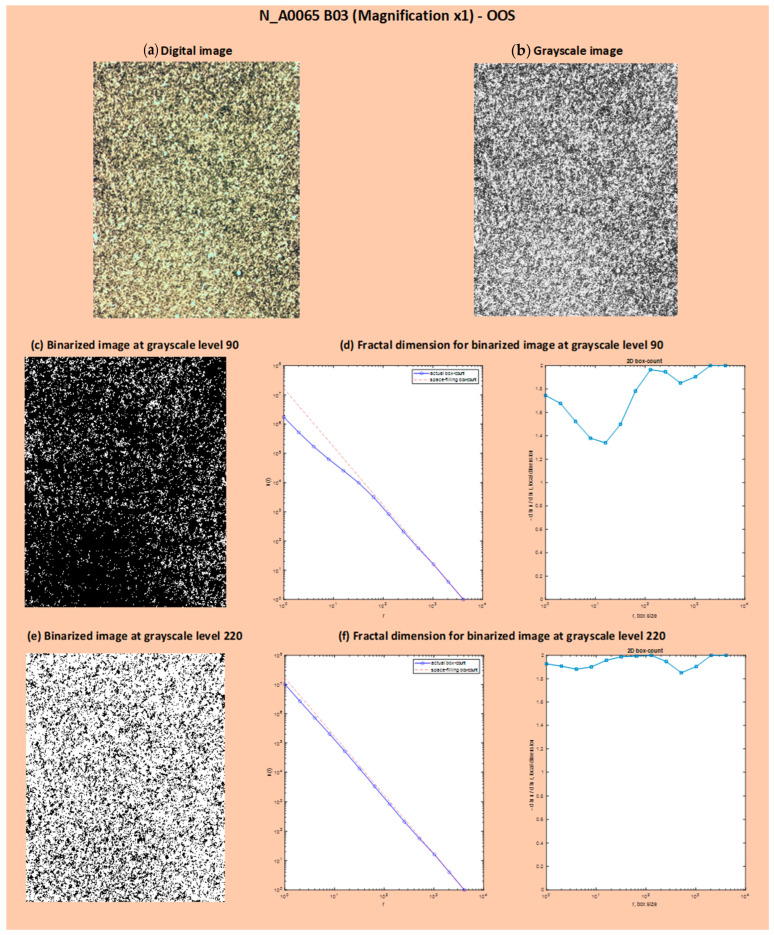
Results of binarization at grayscale levels 90 and 220 and calculation of fractal dimension on the binarized images: OOS sample (N_A0065 B03 at Magnification ×1): digital image (**a**), grayscale image (**b**), binarized image at grayscale level 90 (**c**), fractal dimension for binarized image at grayscale level 90 (**d**), binarized image at grayscale level 220 (**e**) and fractal dimension for binarized image at grayscale level 220 (**f**). In (**d**,**f**), the number of boxes and the local fractal dimension as a function of box size are shown. The red dashed line represents the space-filling curve, which fills a 2D unit box to map the powder sample in the plot. The blue line, the actual box count, aligns the space-filling curve with the scaling exponents measured by the algorithm.

**Figure 12:**
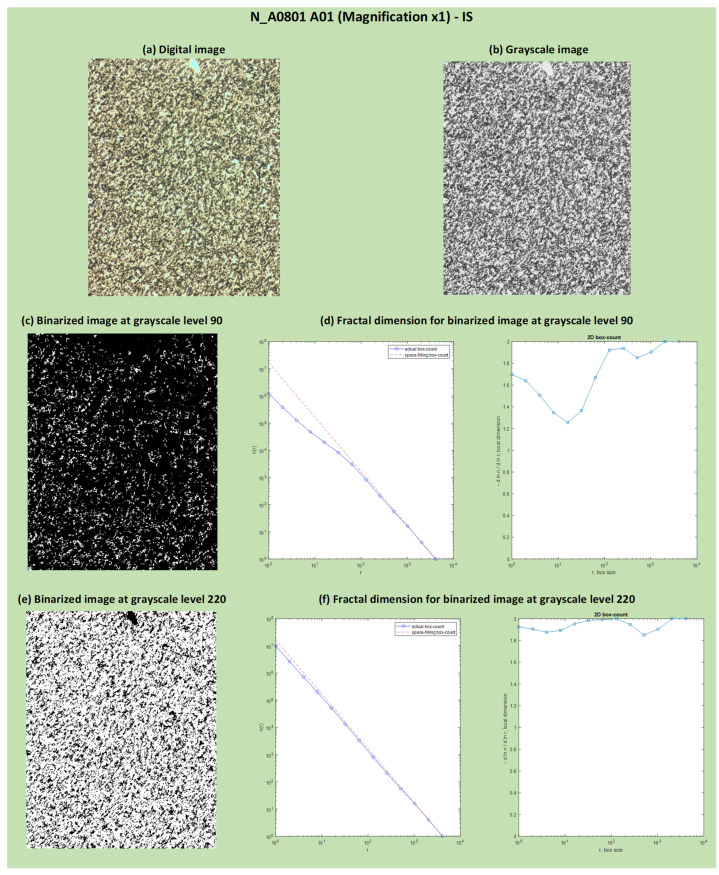
Results of binarization at grayscale levels 90 and 220 and calculation of fractal dimension on the binarized images: IS sample (N_A0801 A01 at Magnification ×1): digital image (**a**), grayscale image (**b**), binarized image at grayscale level 90 (**c**), fractal dimension for binarized image at grayscale level 90 (**d**), binarized image at grayscale level 220 (**e**) and fractal dimension for binarized image at grayscale level 220 (**f**). In (**d**,**f**), the local fractal dimension as a function of box size and the number of boxes as a function of box size are shown. The red dashed line represents the space-filling curve, which fills a 2D unit box to map the powder sample in the plot. The blue line, the actual box count, aligns the space-filling curve with the scaling exponents measured by the algorithm.

**Figure 13:**
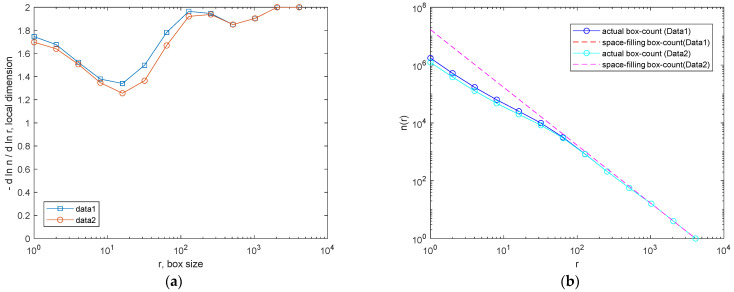
Local fractal dimension as a function of box size (**a**) and number of boxes as a function of box size (**b**) for samples N_A0065 and N_A0801. Data1: N_A0065 B03 (Magnification ×1)—OOS; Data2: N_A0801 A01 (Magnification ×1)—IS. In (**b**), n(r) is the number of boxes, while r represents the box size within the box-counting algorithm. The dashed line represents the space-filling curve, which fills a 2D unit box to map the powder sample in the plot. The continuous line, the actual box count, aligns the space-filling curve with the scaling exponents measured by the algorithm.

As seen in Figure 10, for the identification of OOS samples, it is necessary to analyze binarized images with a grayscale threshold <185. In this section, the results of binarization at a grayscale level of 90 and 220 and the resulting FD dimensions are reported, just as an example, for two digital images: one for the OOS sample (N_A0065 B03 at Magnification ×1; Figure 11) and one for the IS sample (N_A0801 A01 at Magnification ×1; Figure 12). The comparison of local fractal dimension as a function of FD calculation for binarized images at grayscale level 90 for samples N_A0065 B03 (Magnification ×1)—OOS and N_A0801 A01 (Magnification ×1)—IS is shown in Figure 13. As can be observed in Figure 13a, with the same box size (around 102 pixels) and number of boxes, the fractal dimension of the OOS fractal sample is higher than that of the IS sample.

### 3.7. Local Fractal Dimension with a Defined Binarization Threshold (<90 Gray Level)

**Figure 14:**
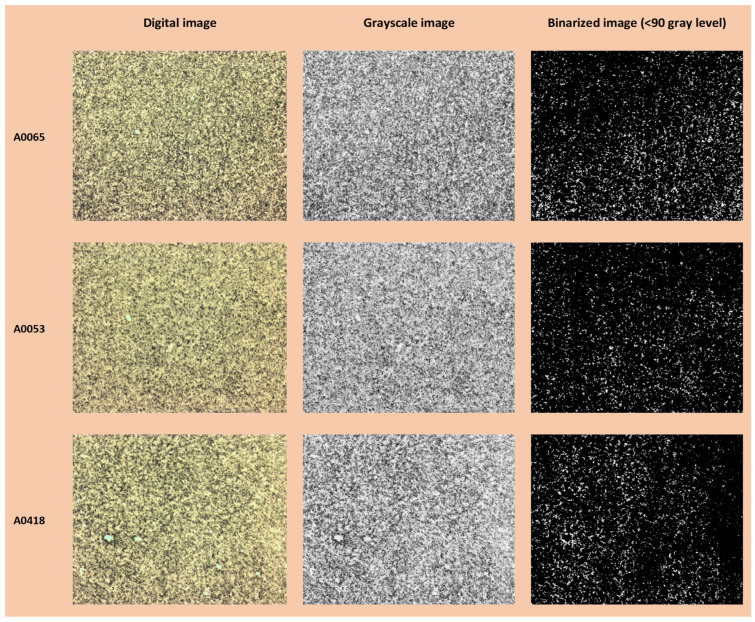
Example: digital image, grayscale image, and binarized image (<90) for OOS samples.

**Figure 15:**
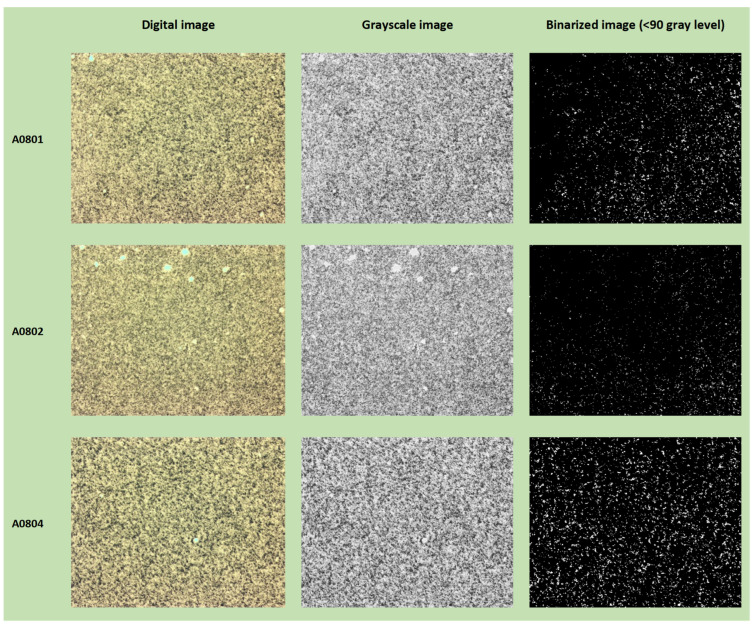
Example: digital image, grayscale image, and binarized image (<90) for IS samples.

**Figure 16:**
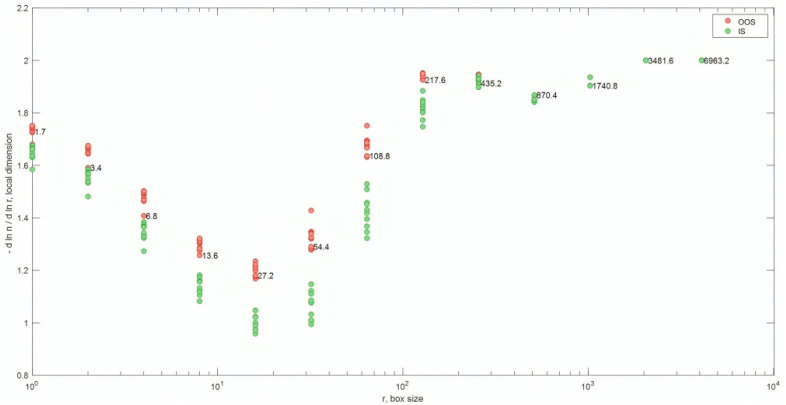
Local fractal dimension as a function of the computation box size for the sample condition. The corresponding computation box sizes in µm are indicated at the local fractal dimension values.

It is important to note that for the same computation box size (around 102 pixels) and number of boxes, the fractal dimension of the OOS sample was greater than that of the IS sample in the test performed with a defined binarization threshold (<90 gray level) for samples N_A0065 and N_A0801 (1× magnification). The calculation of local fractal dimension was performed as a function of the computation box size for all acquired images of samples at 1× magnification and binarized with a gray-level threshold of 90 (Table 2). Figure 14 and Figure 15 show examples of digital image acquisition under the microscope, conversion to grayscale, and binarization with a predefined gray-level threshold of 90 for the OOS samples and IS samples, respectively. The images acquired under the microscope at 1× magnification have a size of 4096 × 3286 pixels. For images acquired at 1× magnification, 1 mm corresponds to 575 ± 10 pixels, so one pixel is approximately 0.0017 mm (1.7 µm). Therefore, the computation boxes for fractal dimension range from 1 pixel to 4096 pixels (1.7–6963 µm). Figure 16 shows the local fractal dimension as a function of the computation box size, calculated for all analyzed samples categorized by sample condition. As can be observed, the local fractal dimension of the OOS samples is slightly higher than that of the IS samples, with the computation box size ranging from 1.7 µm to 217 µm.

### 3.8. Principal Component Analysis of the Local Fractal Dimension with a Defined Binarization Threshold (<90 Gray Level) as a Function of the Computation Box Sizes

**Figure 17:**
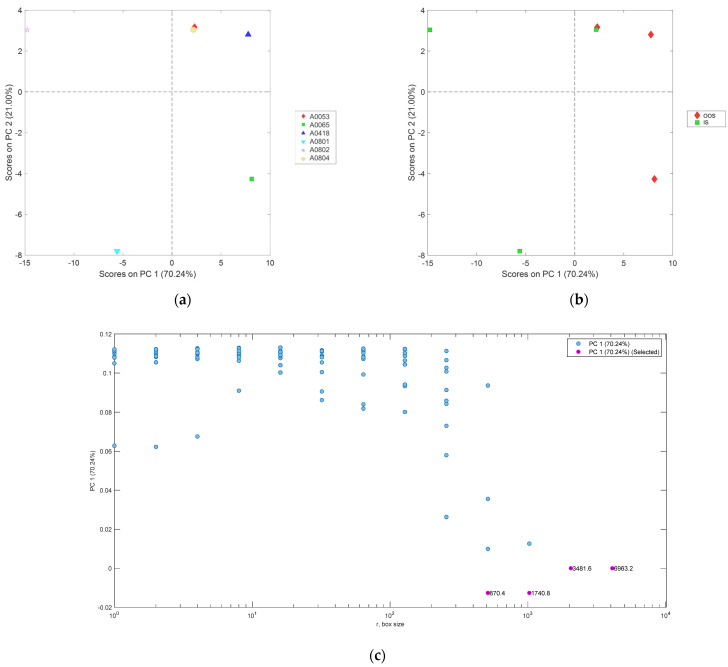
PCA scores plot (PC1 vs. PC2) of the fractal dimension (of the local fractal dimension with defined binarization threshold (<90 gray level) as a function of the calculation box dimensions for samples (**a**) and condition (**b**) and PCA loadings plot (**c**) of the first two principal components with highlighted computation box sizes of 870 µm to 6963 µm.

The arrays of fractal dimension (local fractal dimension with a defined binarization threshold <90 gray level) as a function of the computation box sizes for each analyzed sample at 1× magnification (samples from Table 2), shown in Figure 16, were modeled using principal component analysis (PCA). The results of the PCA are summarized in Figure 17. As highlighted in the scores plot of the first two principal components, the first principal component explains approximately 70% of the variance and successfully discriminates between the OOS and IS groups. Specifically, the scores of the fractal dimension arrays for the OOS samples are in the positive PC1 space, while those of the IS samples are in the negative PC1 space (from computation box sizes of 870 µm to 6963 µm).

## 4. Conclusions

PCAs applied to the PSDs of the analyzed samples showed that the OOS sample scores mainly cluster in a separate space compared to the IS sample scores (Figure 5). The average fractal dimension of the OOS samples is found to be generally higher than the calculated fractal dimension for the IS samples in the <20 and <170 grayscale threshold intervals (Figure 9). Additionally, as highlighted by the PCA, for the purpose of identifying OOS samples, it is necessary to analyze a grayscale threshold of <185 (Figure 10). By setting a threshold value of 90, the local fractal dimension of the OOS samples is slightly higher than that of the IS samples within the calculation box size range of 1.7 µm to 217 µm (Figure 16). From the texture analysis and evaluation of Haralick descriptors (Table 5), it was inferred that:

- The ASM of the OOS samples is generally smaller than that of the IS samples, indicating that the images of the OOS samples are more disordered compared to those of the IS samples.
- The CON parameter of the OOS samples is generally larger than that of the IS samples. This indicates that there is a greater difference in grayscale tones among the pixels in the images of the OOS samples compared to the IS samples.
- The COR parameter is generally lower for the IS samples compared to the OOS samples. This means that in the case of the OOS samples, there is a greater linear dependence of grayscale levels between neighboring pixels in the images compared to the IS samples. The Correlation parameter could, therefore, be an indicator of particle aggregation in the OOS samples.
- Lastly, the ENT parameter is generally higher for the OOS samples compared to the IS samples, indicating that the images of the OOS samples are more disordered compared to the IS samples.
- Based on the results obtained so far, the following hypotheses have been validated:
- The “average” PSD (Particle Size Distribution) of the OOS samples is shifted towards higher values.
- The presence of a portion with a correct PSD and a portion with a coarse PSD causes a general heterogeneity within the batch.

The significant deviation from the expected PSD, coupled with the presence of a bimodal distribution, further underscores the complexity of the particle arrangement in the OOS samples. This collective evidence from multiple analytical techniques corroborates the conclusion that the OOS samples exhibit distinct and anomalous particle size distributions compared to their IS counterparts, highlighting the importance of comprehensive quality control measures in powder production processes.

Finally, the guidance regarding the bimodal nature of the PSD directed the internal investigation of the company’s QC personnel toward identifying all systemic factors that could have caused such a phenomenon. Addressing and resolving these factors led to a significant outcome: the production of over 300 tons of product throughout the entire year of 2023, with minimal occurrences of OOS instances.

The methodology outlined in this paper significantly contributes to ensuring product quality and streamlining manufacturing processes. Moreover, the presented approach enables the analysis of the pseudo-three-dimensional aspect of powder sample images captured under the microscope, facilitating a deeper understanding of particle behavior.

In conclusion, the proposed method offers an insightful and specialized approach to quality control in the analysis of powder particles exhibiting a broad size spectrum. By incorporating fractal and textural analysis, this methodology improves the reliability and accuracy of particle size distribution validation. It identifies irregularities, anomalies, and subtle variations in particle arrangements that traditional measurement techniques may miss. This comprehensive approach enhances the overall quality assessment of powder production processes.

## Figures and Tables

**Table 1:** Amount of material used for acquisition tests with LEICA Wild MZ95™ at magnifications ×1, ×3.2, and ×5. The amount of this material was used to create the N Series samples, a subgroup derived from A0065 (OOS) and A0801 (IS), which are N_A0065 and N_A0801.

Sample ID	Condition	Weight (g)	Weight for Microscopy Analysis (g)
A0801	IS	0.073	0.065
A0065	OOS	0.090	0.084

**Table 2:** Amount of material used for each sample acquired with LEICA Wild MZ95™ at magnification ×1.

Sample ID	Condition	Weight (g)	Weight for Microscopy Analysis (g)
A0053	OOS	0.181	0.170
A0804	IS	0.185	0.174
A0065	OOS	0.202	0.150
A0801	IS	0.185	0.141
A0418	OOS	0.185	0.179
A0802	IS	0.193	0.156

**Table 3:** Average fractal dimension (FD) and standard deviation of the fractal dimension (Std FD) for samples from the N series (N_A0801—IS and N_A0065—OOS) at different magnifications (×5, ×3.2, and ×1) for the different six adopted binarization thresholds (<50, <90, <135, <180, <205, and <230).

Magnification	Sample ID	Sample Condition	Average FD and Std of FD	Grayscale Level (Binarization Threshold)					
				50	90	135	180	205	230
×5	N_A0801	IS	FD	0.690	1.864	1.906	1.923	1.932	1.945
×5	N_A0801	IS	Std FD	0.448	0.107	0.080	0.069	0.064	0.059
×5	N_A0065	OOS	FD	1.122	1.860	1.903	1.922	1.932	1.945
×5	N_A0065	OOS	Std FD	0.344	0.113	0.085	0.070	0.064	0.059
×3.2	N_A0801	IS	FD	0.697	1.860	1.902	1.922	1.932	1.947
×3.2	N_A0801	IS	Std FD	0.358	0.118	0.085	0.071	0.065	0.060
×3.2	N_A0065	OOS	FD	1.102	1.861	1.903	1.922	1.931	1.945
×3.2	N_A0065	OOS	Std FD	0.301	0.115	0.085	0.072	0.066	0.060
×1	N_A0801	IS	FD	1.107	1.794	1.879	1.915	1.931	1.950
×1	N_A0801	IS	Std FD	0.454	0.197	0.117	0.083	0.071	0.063
×1	N_A0065	OOS	FD	1.514	1.805	1.881	1.917	1.933	1.950
×1	N_A0065	OOS	Std FD	0.257	0.172	0.114	0.082	0.070	0.062

**Table 4:** Haralick descriptors—Angular Second Moment (ASM), Contrast (CON), Correlation (COR), and Entropy (ENT)—obtained for the 4 directions (DIR: 0°, 45°, 90°, and 135°) from the co-occurrence matrices of individual digital images at a magnification of ×1 (10 images per sample); subsequently averaged per direction (µ).

	A0065 (OOS)					A0418 (OOS)					A0053 (OOS)				
DIR	0°	45°	90°	135°	µ	0°	45°	90°	135°	µ	0°	45°	90°	135°	µ
ASM	0.110	0.097	0.111	0.097	0.104	0.123	0.109	0.125	0.109	0.117	0.157	0.142	0.158	0.142	0.149
CON	0.201	0.266	0.192	0.266	0.231	0.172	0.227	0.161	0.227	0.197	0.145	0.196	0.142	0.196	0.170
COR	0.963	0.951	0.965	0.951	0.957	0.967	0.956	0.969	0.956	0.962	0.962	0.949	0.963	0.949	0.956
ENT	3.524	3.683	3.499	3.683	3.597	3.418	3.575	3.386	3.575	3.489	3.156	3.314	3.147	3.314	3.233
	**A0802 (IS)**					**A0804 (IS)**					**A0801 (IS)**				
**DIR**	**0°**	**45°**	**90°**	**135°**	**µ**	**0°**	**45°**	**90°**	**135°**	**µ**	**0**	**45°**	**90°**	**135°**	**µ**
ASM	0.180	0.164	0.181	0.164	0.172	0.151	0.137	0.153	0.137	0.144	0.172	0.156	0.173	0.156	0.164
CON	0.139	0.186	0.136	0.186	0.162	0.148	0.198	0.142	0.198	0.171	0.151	0.200	0.146	0.200	0.174
COR	0.951	0.934	0.952	0.934	0.943	0.963	0.951	0.965	0.951	0.957	0.952	0.937	0.954	0.937	0.945
ENT	2.943	3.093	2.933	3.093	3.016	3.192	3.344	3.172	3.344	3.263	3.037	3.184	3.020	3.184	3.106

**Table 5:** Haralick descriptors—Angular Second Moment (ASM), Contrast (CON), Correlation (COR), and Entropy (ENT)—obtained for the 4 directions (DIR: 0°, 45°, 90°, and 135°) from the co-occurrence matrices of individual digital images at a magnification of ×1, averaged by condition (IS or OOS); subsequently averaged per direction (µ).

	OOS					IS				
DIR	0°	45°	90°	135°	µ	0°	45°	90°	135°	µ
ASM	0.130	0.116	0.131	0.116	0.123	0.167	0.152	0.169	0.152	0.16
CON	0.173	0.23	0.165	0.23	0.199	0.146	0.195	0.141	0.195	0.169
COR	0.964	0.952	0.966	0.952	0.958	0.955	0.941	0.957	0.941	0.948
ENT	3.366	3.524	3.344	3.524	3.439	3.058	3.207	3.041	3.207	3.128

## Data Availability

The data used in this study is available from the corresponding author upon reasonable request. The MATLAB scripts and functions used for fractal dimension as a function of the grayscale level used as the binarization threshold are available at GitHub: https://github.com/Gasby90/FD_multiple_images_grayscale_level (accessed on 20 November 2023 Month Year). The MATLAB script used to perform textural analysis on multiple images is available at GitHub: https://github.com/Gasby90/textural_multiple_images (accessed on 20 November 2023).

## Appendix A

Table A1 reports the percentiles of the particle size distributions (PSDs) obtained by the laser diffraction method. Dv(10), Dv(50), and Dv(90) represent specific percentiles of the cumulative PSD. In more detail, Dv(10) indicates the particle diameter below which 10% of the sample volume exists; Dv(50), also known as the median particle size by volume, represents the particle diameter below which 50% of the sample volume exists. This parameter corresponds to the midpoint of the cumulative particle size distribution, where half of the particles by volume are smaller, and half are larger. Finally, Dv(90) is the particle diameter below which 90% of the sample volume exists.

**Table A1:** Percentiles of the particle size distributions (PSDs) obtained by laser diffraction method: Dv(10), Dv(50), and Dv(90).

Sample ID	A0053	A0065	A0418	A0801	A0802	A0804
Condition	OOS	OOS	OOS	IS	IS	IS
Dv(10)	5.4 µm	5.9 µm	4.7 µm	4.2 µm	3.9 µm	4.9 µm
Dv(50)	15.6 µm	16.1 µm	13.4 µm	12.1 µm	11.2 µm	13.7 µm
Dv(90)	41.1 µm	40.1 µm	34.8 µm	29.0 µm	26.9 µm	34.8 µm

## Appendix B. (Additional Information)

Detailed information about the MATLAB function boxcount.m by Frederic Moisy can be retrieved at: https://it.mathworks.com/matlabcentral/fileexchange/13063-boxcount (accessed on 20 November 2023).

Detailed information about the MATLAB function HaralickTextureFeatures.m by Rune Monzel can be retrieved at: https://www.mathworks.com/matlabcentral/fileexchange/58769-haralicktexturefeatures (accessed on 20 November 2023).
